# Percutaneous coronary interventions improve phasic cardiac–coronary interaction in chronic coronary syndromes as assessed by wave intensity analysis

**DOI:** 10.1136/openhrt-2026-004194

**Published:** 2026-07-21

**Authors:** Ahmet Taş, Yaren Alan, Alp Ozcan, Ilke Kara Taş, Nehir Taş, Irem Sezer, Tim P van de Hoef, Kim H. Parker, Murat Sezer, Jan J Piek

**Affiliations:** 1 Cardiovascular Sciences, Amsterdam UMC Locatie AMC, Amsterdam, Netherlands; 2 Department of Cardiology, Istanbul Mehmet Akif Ersoy Thoracic and Cardiovascular Surgery Training and Research Hospital, Istanbul, Turkey; 3 Department of Cardiology, Koc University School of Medicine, Istanbul, Turkey; 4 Department of Pathology, Istanbul Training and Research Hospital, Istanbul, Turkey; 5 Istanbul Medipol University International School of Medicine, Beykoz, Turkey; 6 National Amyloidosis Centre, Royal Free Hospital, London, UK; 7 Cardiology, University Medical Centre Utrecht, Utrecht, Netherlands; 8 Department of Bioengineering, Imperial College London, London, UK; 9 Department of Cardiology, Acıbadem International Hospital, Istanbul, Turkey

**Keywords:** Percutaneous Coronary Intervention, Coronary Vessels, Coronary Artery Disease

## Abstract

**Background:**

Clinical benefit of percutaneous coronary intervention (PCI) in chronic coronary syndromes (CCS) remains controversial. Coronary wave intensity analysis (WIA) provides mechanistic insights into cardiac–coronary coupling.

**Objectives:**

To assess peri-PCI WIA changes at rest and during hyperaemia, and to identify features associated with improvements in epicardial and microvascular resistance.

**Methods:**

Intracoronary Doppler and pressure measurements were analysed in 27 CCS patients undergoing elective PCI. Haemodynamics (fractional flow reserve (FFR), basal and hyperaemic stenosis resistance (bSR, hSR), coronary flow reserve (CFR), basal and hyperaemic microvascular resistance (bMR, hMR)) and WIA metrics (forward compression wave (FCW), backward compression wave (BCW), forward expansion wave (FEW), backward expansion wave (BEW), wave speed) were quantified at rest and hyperaemia.

**Results:**

PCI reduced bSR and hSR (p<0.001), increased FFR (0.58±0.18 to 0.87±0.09; p<0.001) and CFR (1.68±0.47 to 2.79±0.62; p<0.001), and lowered hMR (2.22 (1.67–3.10) to 1.49 (1.18–1.74); p<0.001). At rest, PCI increased FCW (40.3±48.9 to 89.3±64.8; p=0.022), FEW (24.5 (7.2–42.8) to 58.8 (24.0–93.1); p=0.001) and BEW (57.6±70.6 to 82.1±51.8; p=0.017). Hyperaemic backward wave peaks augmented significantly (BCW: 77.8±53.3 to 172.8±112.3; BEW: 60.5±47.5 to 126.7±66.2; both p<0.001). WIA time-to-peaks occurred earlier post-PCI. Reduced resting wave speed (41±21 to 25±10 m/s; p<0.001) correlated with hMR decreases (r=0.77; p<0.001). Increased resting BEW correlated with total vascular resistance reductions (hSR+hMR; r=−0.76; p<0.001).

**Conclusions:**

Elective PCI restores phasic cardiac–coronary coupling. Resting BEW and coronary wave speed provide complementary physiological endpoints reflecting microvascular and epicardial recovery, warranting larger prospective evaluation.

**WHAT IS ALREADY KNOWN ON THIS TOPIC**

**WHAT THIS STUDY ADDS**

- This study demonstrates that elective PCI restores phasic energy transfer by significantly augmenting forward and backward expansion waves and normalising ventricular–coronary interaction delays. Notably, post-PCI reductions in estimated coronary wave speed and increases in resting backward expansion waves correlate strongly with vascular resistance improvements.

**HOW THIS STUDY MIGHT AFFECT RESEARCH, PRACTICE OR POLICY**

- These findings suggest that wave intensity-derived metrics can serve as complementary physiological endpoints to assess the mechanistic success of PCI beyond standard pressure-only indices. This approach may help identify patients with residual haemodynamic perturbations despite epicardial revascularisation.

## Introduction

 In chronic coronary syndromes (CCS), the reduction in perfusion pressure induced by obstructive coronary artery disease (CAD) is compensated by an adaptive response of the coronary microcirculation that influences microvascular haemodynamic parameters. Coronary wave intensity analysis (WIA) of simultaneous pressure and flow measurements quantifies the phasic arterial wave energy transfer that establishes coronary flow. Unlike pressure-only indices, WIA is not intended as a binary revascularisation tool; rather, it characterises the phasic pressure-flow forces that govern coronary filling and therefore complements lesion-level decision indices. The characterisation of the expansion versus compression and accelerating versus decelerating nature of the waves reveals haemodynamic alterations of the epicardial conduit artery and microvascular bed. In CCS, depending on the extent of the microvascular compensation, the impact of an epicardial narrowing on the WIA profile may vary among cardiac patients. Previous research reported contradicting relationships between the severity of epicardial disease and WIA parameters.^1 2^ Moreover, the impact of the relief of coronary obstruction by percutaneous coronary intervention (PCI) on WIA was not fully elucidated. The aim of this study is to comprehensively describe the effect of PCI on the coronary WIA profile and identify potential WIA parameters associated with more favourable post-PCI haemodynamics, including improvement of epicardial resistance and microvascular decompression.

## Methods

The present study reports a retrospective analysis of invasive coronary physiology recordings obtained during elective PCI in patients with CCS at Amsterdam UMC (Heart Centre, Amsterdam, the Netherlands). Adults with stable angina and a single target epicardial stenosis scheduled for elective PCI were eligible. Patients with left main coronary artery stenosis, diffuse disease, serial lesions, subtotal lesions (defined as angiographic near-occlusion with ≥90% diameter stenosis and/or impaired antegrade flow (TIMI≤2)) in the target vessel, or severe renal dysfunction were excluded. History of PCI was not an exclusion criterion, provided an adequate target-vessel pressure–Doppler recording before and after the index PCI was available. This dataset was retrieved from the institutional repository following IRB approval. A total of 29 patients with an indication for elective PCI (not enrolled in prospective trials) and available paired pre- and post-PCI measurements were identified from 2007 to 2008. Two were excluded because of inadequate-quality velocity traces, leaving 27 patients for analysis. Aortic pressure (Pa) was measured via a guiding catheter placed in the coronary ostium (standard femoral approach). Distal pressure (Pd) and Doppler flow velocity were measured using a 0.014-inch guidewire equipped with both a Doppler velocity probe and a pressure sensor (Combowire, Volcano, San Diego, California, USA). The wire was positioned in the target vessel with both sensors located distally to the coronary stenosis. All haemodynamic signals were recorded at 120 Hz. Measurements were performed at rest and during adenosine-induced hyperaemia according to institutional routine. No external core laboratory was used; all signal processing and WIA were performed offline by investigators blinded to the PCI timepoint.

### Definitions of haemodynamic indices

Basal (bMR) and hyperaemic (hMR) microvascular resistances were calculated using mean distal coronary pressure (Pd) and average peak velocity (APV) as bMR=Pd_rest/APV_rest and hMR=Pd_hyp/APV_hyp (mm Hg/s/cm). Baseline (bSR) and hyperaemic stenosis resistance (hSR) were defined as bSR=(Pa_rest−Pd_rest)/APV_rest and hSR=(Pa_hyp−Pd_hyp)/APV_hyp (mm Hg/s/cm). The fractional flow reserve (FFR) was calculated as FFR=Pd_hyp/Pa_hyp during hyperaemia. Coronary flow reserve (CFR) was calculated as CFR=APV_hyp/APV_rest. Resistance reserve ratio (RRR) was defined as RRR=bMR/hMR. Microvascular resistance reserve (MRR) was calculated as MRR=(CFR/FFR)×(Pa_rest/Pa_hyp). Finally, total coronary artery resistance during hyperaemia was calculated as (hSR+hMR).

### Analysis of haemodynamic signals

**Figure 1:**
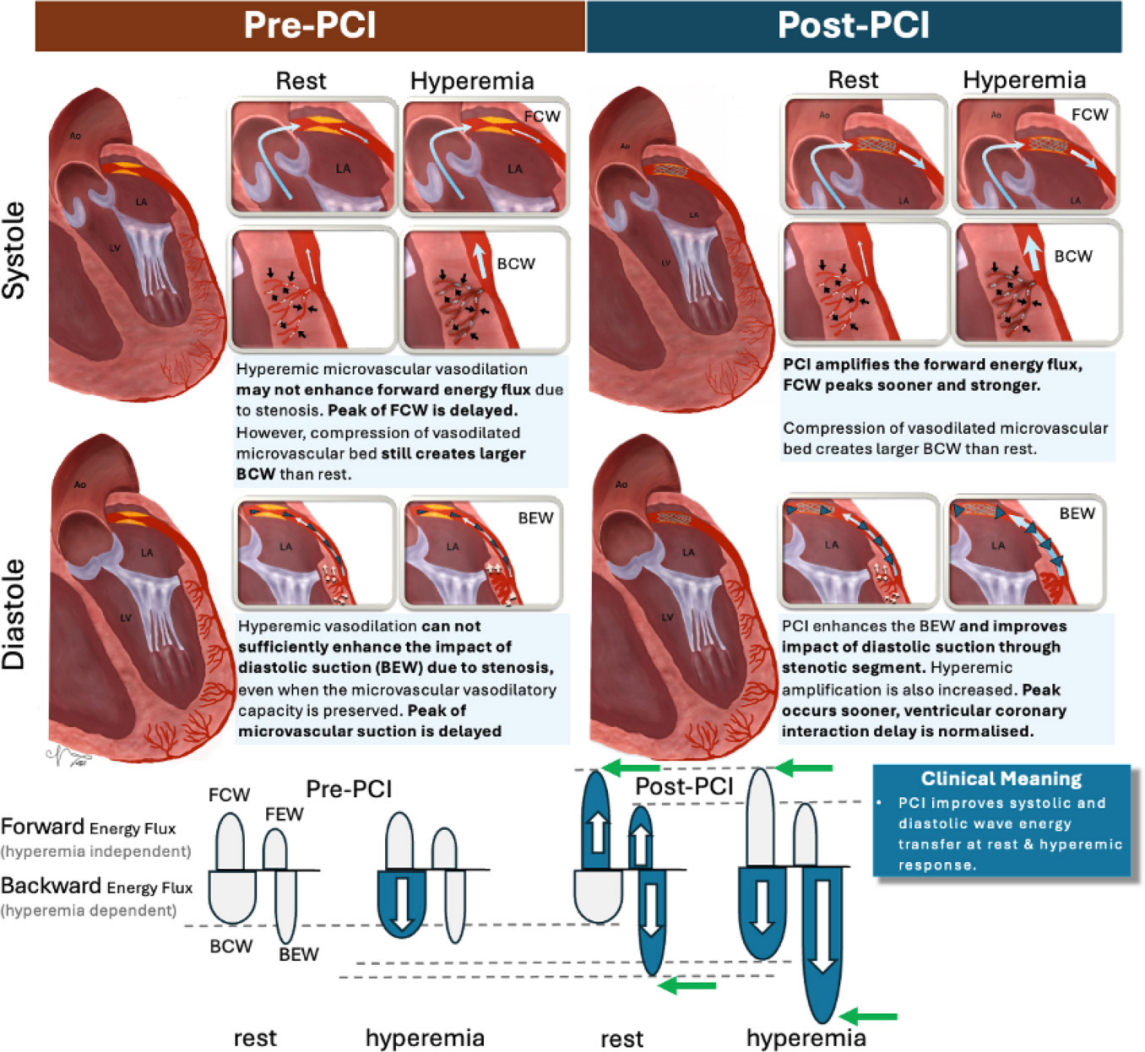
(Visual Abstract) Impact of PCI on coronary wave energy flux. Elective PCI normalises and improves cardiac–coronary coupling. Ao, aorta; BCW, backward compression wave; BEW, backward expansion wave; FCW, forward compression wave; FEW, forward expansion wave; LA, left atrium; LV, left ventricle; PCI, percutaneous coronary intervention.

Invasively obtained Doppler coronary (flow) velocity and pressure data recorded at rest and during hyperaemia were imported into MATLAB (v2024a, MathWorks) to visualise and quantify the flow, pressure and resistance-based indices of epicardial and microvascular disease severity as well as the coronary WIA. The wave-intensity analysis used to quantitatively evaluate the coronary arterial energy transfer characteristics was performed with an updated version of Kim H. Parker’s dedicated software (Imperial College, UK)^3^ used in our previous studies.^4^ Net wave intensity (WI) was computed with arterial blood pressure and velocity signals as previously described (WI=(dP/dt)/(dU/dt), kW/m^2^/s^2^) figure 1. The sampling rate was 120/s and derivatives were calculated using a second-order Savitsky-Golay filter with a window length of 11 samples. Wave separation and estimation of wave speed (c) were performed using the sum-of-squares method^3 5^ (Σ²): c=(1/ρ)/√(Σ(dP)²/Σ(dU)²), where ρ is blood density (assumed 1060 kg/m³) and the summation is taken over the widest temporal frame with adequate signal quality (≥8–10 consecutive beats). Variables of interest were peak amplitudes of the forward compression (FCW), backward compression (BCW), forward expansion (FEW) and backward expansion (BEW; diastolic suction wave) waves.^6 7^ The accelerating wave energy proportion (AEP%) was defined as AEP%=(FCW+BEW)/(FCW+BCW+FEW+BEW). Relative time to peaks of four major waves was assessed and defined as previously described, where higher values indicated later peaks.^7^

### Statistical analysis

Continuous variables are expressed as mean±SD for normally distributed data. The normality of paired differences was assessed using the Shapiro-Wilk test. Variables that violated the assumption of normality are presented as the median (25th–75th percentiles (IQR)). ∆ indicates the change between pre- and post-PCI state for a given variable, and δ indicates the change from rest to hyperaemia. Pre- versus post-PCI comparisons at rest and during hyperaemia were evaluated using paired t-tests for normally distributed data and non-parametric Wilcoxon signed-rank tests for skewed data. Correlations between changes in WIA metrics and changes in resistance indices were evaluated using Pearson’s or Spearman’s correlation, as appropriate based on data distribution. Given the large number of correlations and paired tests evaluated, we designated the associations between ∆BEW, ∆wave-speed and the normalisation of resistance parameters as primary hypotheses based on physiological rationale. No a priori sample size calculation was performed because this was a retrospective, hypothesis-generating study; however, based on the observed within-patient variability, a future prospective study would require approximately 39 patients to detect the observed change in resting BEW with 80% power at α=0.05. All analyses were performed using the R-based Jamovi statistical software package.

## Results

### Demographic characteristics

Study population (N=27) mainly included male patients (74%) and the mean age was 59±8 years. Diabetes (26%), hypertension (33%) and current smoking (74%) were the most prevalent cardiovascular risk factors. Mean angiographic diameter stenosis % pre-PCI was 66.5%±16.9%.

### Impact of PCI on coronary haemodynamics and WIA

**Figure 2:**
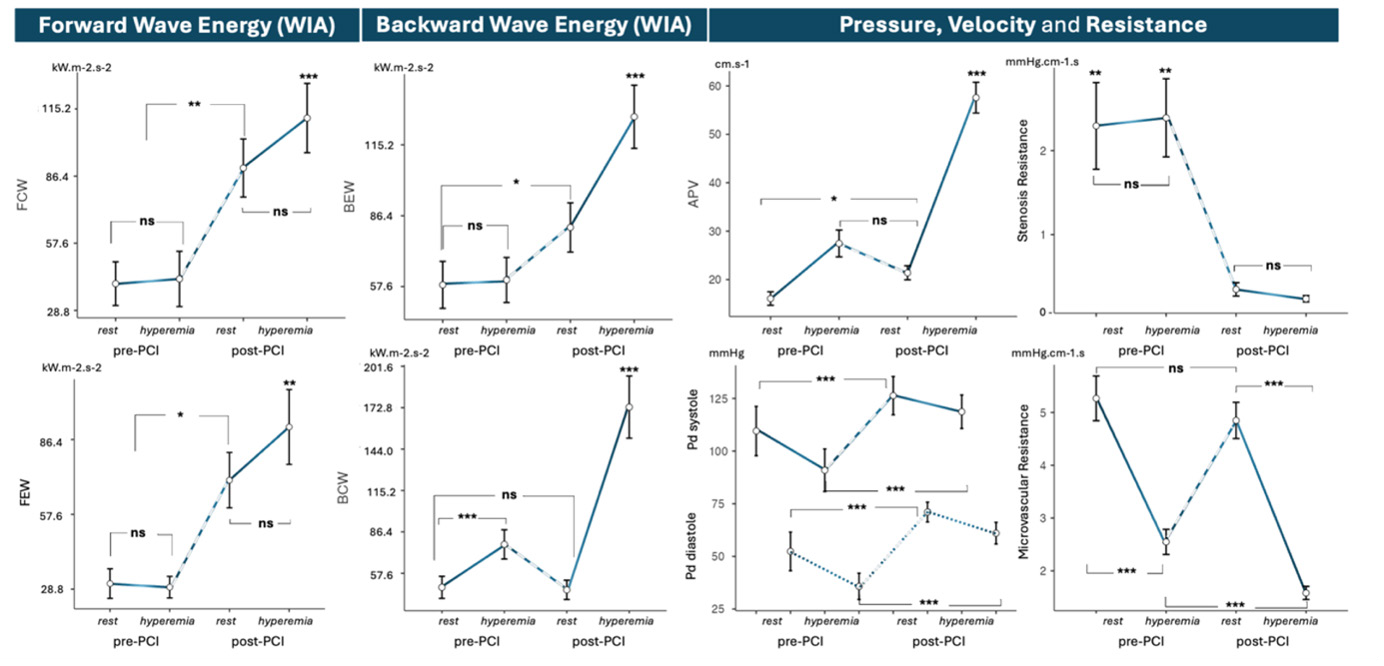
Peri-PCI alterations of coronary haemodynamics and WIA peaks at rest and during hyperaemia. Note that the y axes are different to better display the pairwise comparisons, zero point is suppressed in most plots. Values provided as mean (SE of means) ***p<0.001; **p<0.01; *p<0.05. APV, average peak velocity; BCW, backward compression wave; BEW, backward expansion wave; FCW, forward compression wave; FEW, forward expansion wave; PCI, percutaneous coronary intervention; WIA, wave intensity analysis.

PCI was associated with a substantial reduction in stenosis resistance at rest (bSR 1.13 (0.58–2.57) to 0.22 (0.12–0.39) mm Hg/s/cm, p<0.001) and during hyperaemia (hSR 1.34 (0.69–3.09) to 0.16 (0.11–0.37) mm Hg/s/cm, p<0.001) (figure 2, table 1). Distal coronary pressure and flow velocity increased, resulting in higher FFR (0.58±0.18 to 0.87±0.09, p<0.001) and CFR (1.68±0.47 to 2.79±0.62, p<0.001). Hyperaemic microvascular resistance decreased (2.22 (1.67–3.10) to 1.49 (1.18–1.74) mm Hg/s/cm, p<0.001), while basal microvascular resistance was not significantly changed (4.49 (3.89–6.61) to 4.79 (3.85–5.82) mm Hg/s/cm, p=0.374). At rest, PCI significantly augmented the forward travelling waves: FCW (40.3±48.9 vs 89.3±64.8, p=0.022) and FEW (24.5 (7.2–42.8) vs 58.8 (24.0–93.1) kW/m²/s², p=0.001). The backward expansion wave (BEW) also increased at rest (57.6±70.6 vs 82.1±51.8 kW/m²/s², p=0.017), whereas the resting BCW amplitude remained similar (p=0.934).

**Table 1:** Impact of PCI on coronary haemodynamics and WIA

Measure	N	Pre**-**PCI (mean±SD or Median (IQR))	Post**-**PCI (mean±SD or Median (IQR))	Shapiro-Wilk p value	Comparison p value
bAPV (cm/s**)**	27	16.1±7.2	21.4±7.5	0.130	0.010
hAPV (cm/s**)**	27	27.5±14.4	57.6±17.7	0.295	<0.001
bMR (mm Hg/s/cm)	27	4.49 (3.89–6.61)	4.79 (3.85–5.82)	0.046	0.374
hMR (mm Hg/s/cm)	27	2.22 (1.67–3.10)	1.49 (1.18–1.74)	0.002	<0.001
bSR (mm Hg/s/cm)	27	1.13 (0.58–2.57)	0.22 (0.12–0.39)	<0.001	<0.001
hSR (mm Hg/s/cm)	27	1.34 (0.69–3.09)	0.16 (0.11–0.37)	<0.001	<0.001
FFR (unitless)	27	0.58±0.18	0.87±0.09	0.200	<0.001
Pd/Pa (unitless)	27	0.74±0.21	0.93±0.07	0.086	<0.001
CFR (unitless)	27	1.68±0.47	2.79±0.62	0.893	<0.001
RRR (unitless)	27	2.21±0.66	3.14±0.83	0.420	<0.001
MRR (unitless)	27	3.10±0.72	3.39±0.92	0.135	0.055
FCW rest (kW/m²/s²)	27	40.3±48.9	89.3±64.8	0.258	0.022
BCW rest (kW/m²/s²)	27	47.5±40.3	46.1±34.6	0.058	0.934
FEW rest (kW/m²/s²)	27	24.5 (7.2–42.8)	58.8 (24.0–93.1)	0.026	0.001
BEW rest (kW/m²/s²)	27	57.6±70.6	82.1±51.8	0.796	0.017
AEP rest (**%**)	27	54±10	59±8	0.220	0.010
FCW hyp (kW/m²/s²)	27	41.8±61.9	110.9±76.4	0.120	<0.001
BCW hyp (kW/m²/s²)	27	77.8±53.3	172.8±112.3	0.070	<0.001
FEW hyp (kW/m²/s²)	27	24.0 (11.1–43.8)	61.3 (29.9–118.1)	0.007	<0.001
BEW hyp (kW/m²/s²)	27	60.5±47.5	126.7±66.2	0.871	<0.001
AEP hyp (**%**)	27	48 (39–56)	45 (42–51)	0.047	0.485

Uncorrected p values are reported. Comparisons other than the primary hypotheses are exploratory.

AEP, accelerating wave energy proportion; bAPV, baseline average peak velocity; BCW, backward compression wave; BEW, backward expansion wave; bMR, basal microvascular resistance; bSR, basal stenosis resistance ; CFR, coronary flow reserve; FCW, forward compression wave; FEW, forward expansion wave; FFR, fractional flow reserve; hAPV, hyperaemic average peak velocity; hMR, hyperaemic microvascular resistance; hMR, hyperaemic microvascular resistance; hSR, hyperaemic stenosis resistance ; hSR, hyperaemic stenosis resistance; MRR, microvascular resistance reserve; PCI, percutaneous coronary intervention; RRR, resistance reserve ratio .

During hyperaemia, both backward travelling waves (BCW 77.8±53.3 vs 172.8±112.3 and BEW 60.5±47.5 vs 126.7±66.2 kW/m²/s²; both p<0.001) and forward travelling waves (FCW 41.8±61.9 vs 110.9±76.4; FEW 24.0 (11.1–43.8) vs 61.3 (29.9–118.1) kW/m²/s²; both p<0.001) augmented significantly. PCI also increased the proportion of accelerating wave energy flux at rest (AEP% 54±10 vs 59±8%, p=0.010), whereas AEP% during hyperaemia did not change (48 (39–56) vs 45 (42 – 51) %, p=0.485). MRR showed a trend towards improvement but did not reach significance (3.10±0.72 vs 3.39±0.92, p=0.055).

**Figure 3:**
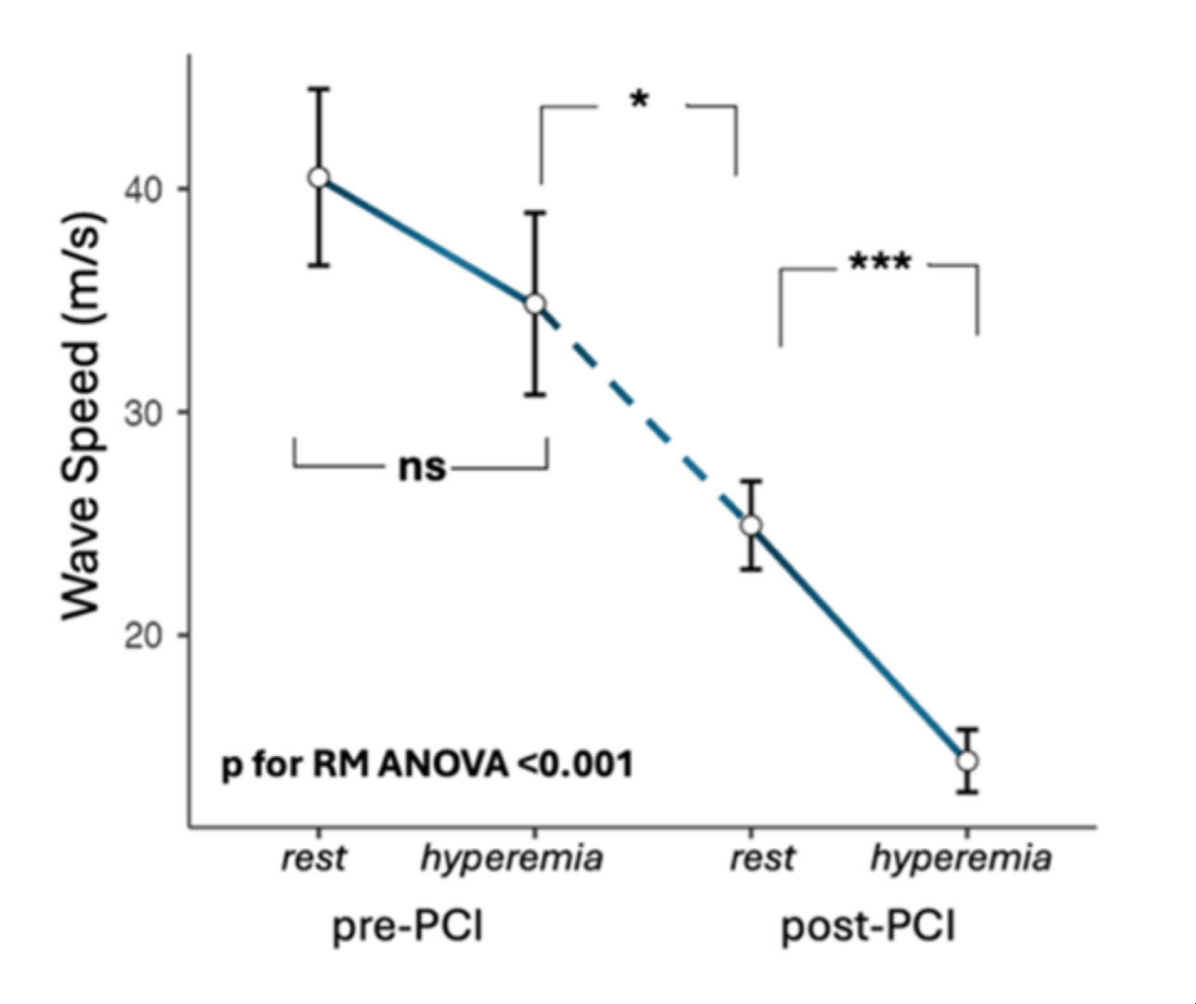
PCI normalises the elevated pre-PCI coronary wave-speed. (Pre-PCI rest=41±21 m/s, hyperaemia=35±21 m/s. Post-PCI rest=25±10 m/s, hyperaemia=14±7 m/s.) PCI, percutaneous coronary intervention; RM ANOVA, repeated measures analysis of variance. Data is expressed as mean [SE of means].

**Figure 4:**
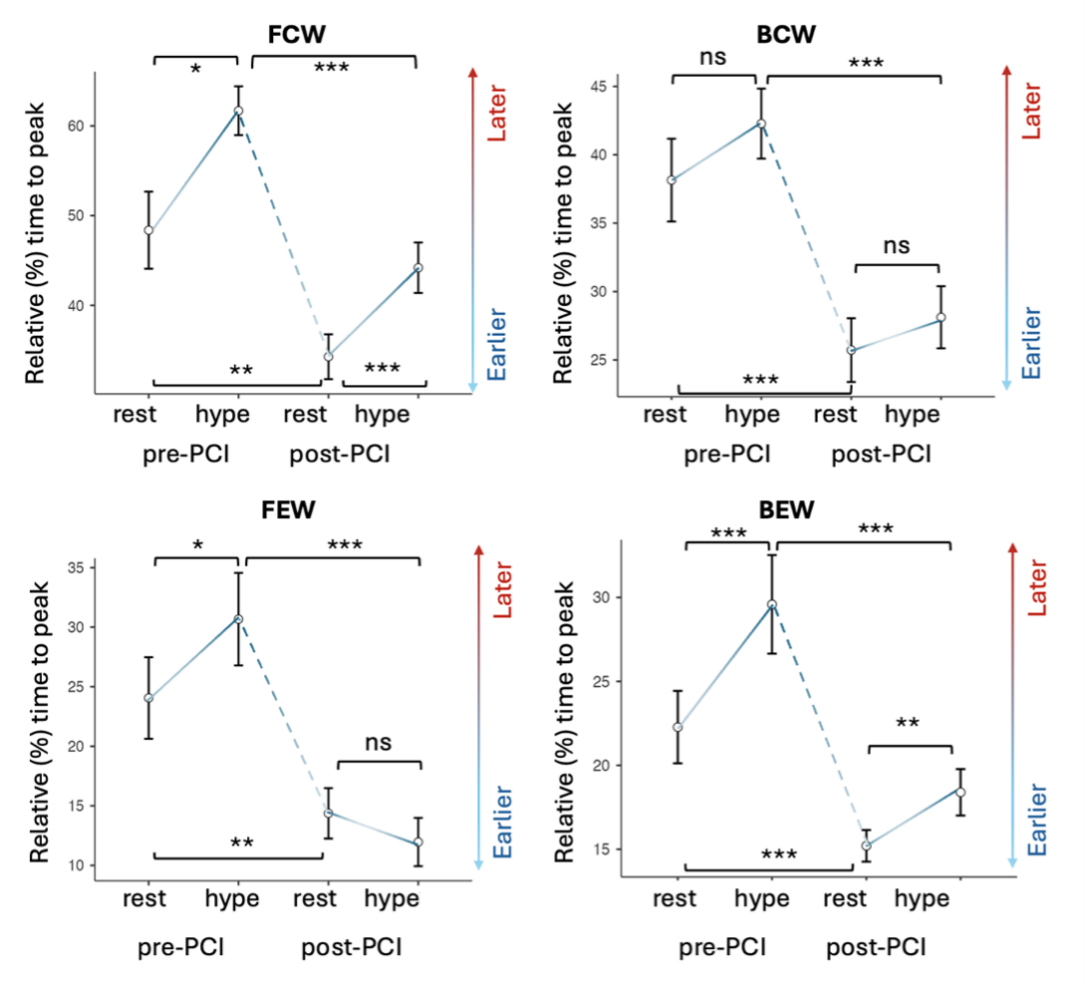
Peri-PCI alterations of relative time to peaks of wave-intensity at rest and during hyperaemia. PCI improved ventricular–coronary interaction delay with the removal of epicardial stenoses. Note that the y axes are different to better display the pairwise comparisons, zero point is suppressed in most plots. Values provided as mean (SE of means). ***p<0.001; **p<0.01; *p<0.05. BCW, backward compression wave; BEW, backward expansion wave; FCW, forward compression wave; FEW, forward expansion wave; PCI, percutaneous coronary intervention.

Calculated coronary wave speed decreased after PCI both at rest (41±21 vs 25±10 m/s) and during hyperaemia (35±21 vs 14±7 m/s) (both p<0.001) (figure 3). Time-to-peak of all major wave components occurred earlier post-PCI at rest and during hyperaemia, indicating normalisation of ventricular–coronary interaction delay (all p<0.05) (figure 4). Magnitude of this improvement correlated with the normalisation of epicardial patency and resistance.

### Relationship between changes in epicardial and microvascular resistances and WIA

**Figure 5:**
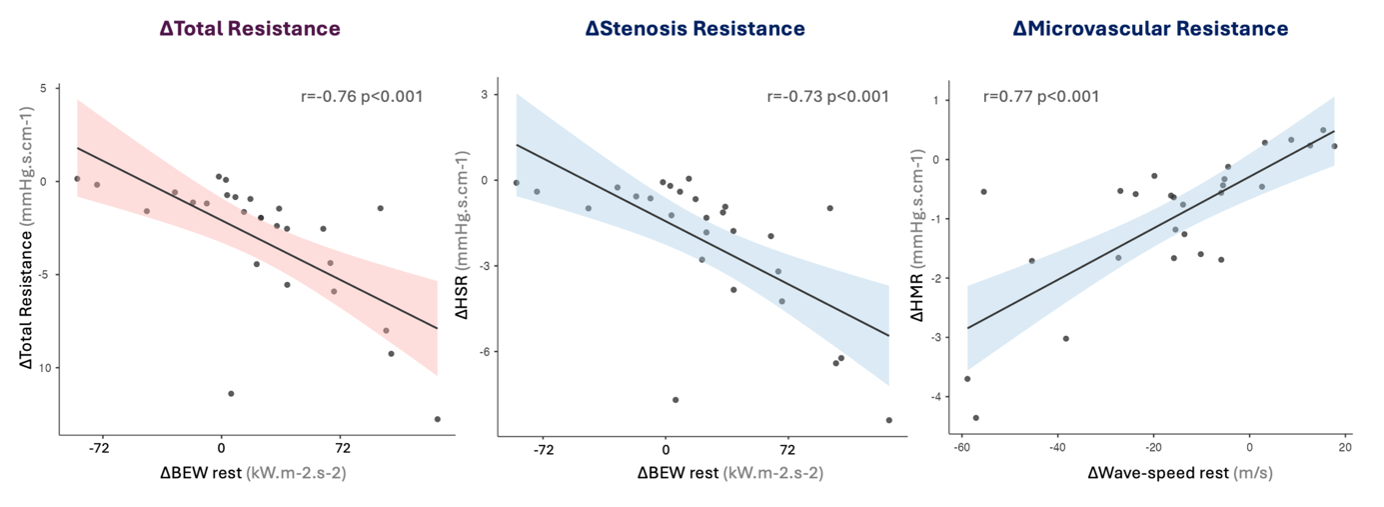
Associations between changes in WIA metrics and resistance indices. Increase in resting BEW was associated with a larger reduction in total coronary resistance (hSR+hMR) and stenosis resistance (hSR), while attenuation of resting wave speed was associated with lower values of hyperaemic microvascular resistance (hMR). BEW, backward expansion wave; WIA, wave intensity analysis.

Change in resting BEW amplitude (∆BEW_rest) between pre- and post-PCI was strongly associated with attenuation of total coronary resistance. A larger increase in resting BEW was associated with a larger reduction in total coronary resistance (∆(hSR+hMR); r=−0.76, p<0.001) and stenosis resistance (∆hSR; r=−0.73, p<0.001). Reduction in resting wave speed was associated with a reduction in hyperaemic microvascular resistance (∆hMR; r=0.77, p<0.001) (figure 5; Table 2).

**Table 2:** Correlations between magnitudes of peri-percutaneous coronary intervention changes

		∆bMR	∆hMR	∆bSR	∆hSR	∆TVR	∆FFR
Rest	∆FCW_peak_	−0.587**	−0.498**	−0.469*	−0.531**	−0.607***	0.515**
	∆BCW_peak_	−0.632***	−0.570**	−0.413*	−0.444*	−0.580**	0.438*
	∆FEW_peak_	−0.439*	−0.356	−0.574**	−0.599**	−0.551**	0.676***
	∆BEW_peak_	−0.643***	−0.597**	−0.699***	−0.732***	−0.762***	0.719***
	∆AEP%	−0.323	−0.333	−0.342	−0.369	−0.386*	0.194
	∆tFCW_peak_	0.326	0.444*	0.420*	0.495*	0.469*	−0.463*
	∆tBEW_peak_	0.189	0.497*	0.577**	0.558**	0.544**	−0.376
Hyperaemia	∆FCW _peak_	−0.550**	−0.400*	−0.372	−0.419*	−0.502**	0.496**
	∆BCW _peak_	−0.447*	−0.537**	−0.346	−0.404*	−0.518**	0.346
	∆FEW _peak_	−0.226	−0.162	−0.330	−0.377	−0.314	0.577**
	∆BEW _peak_	−0.468*	−0.421*	−0.395*	−0.426*	−0.471*	0.465*
	∆AEP%	0.032	0.053	0.129	0.147	0.110	−0.214
	∆tFCW_peak_	0.250	−0.084	−0.089	−0.002	−0.024	−0.106
	∆tBEW_peak_	0.215	0.285	0.006	0.127	0.224	0.013

*** indicates p<0.001 ; ** indicates p<0.01; * indicates p<0.05

AEP, accelerating wave energy proportion; BCW, backward compression wave; BEW, backward expansion wave; bMR, basal microvascular resistance; bSR, basal stenosis resistance; FCW, forward compression wave; FEW, forward expansion wave; FFR, fractional flow reserve; hMR, hyperaemic microvascular resistance; hSR, hyperaemic stenosis resistance; TVR, total vascular resistance.

## Discussion

In this study, we have demonstrated the distinct peri-PCI changes in WIA parameters reflecting improvement of cardiac–coronary coupling, together with PCI-induced reduction of total vascular resistance, indicating the mechanistic relevance of WIA in the PCI setting. The key messages are as follows:

1. PCI improves systolic and diastolic cardiac–coronary coupling under resting and hyperaemic conditions cumulatively by normalisation of epicardial and total coronary resistance, decompression of microvascular bed, normalisation of ventricular–coronary interaction delay and improved responsiveness to hyperaemia as determined by coronary WIA.
2. A sooner and stronger peak of resting BEW following PCI compared with pre-PCI state, indicating a normalised microvascular suction through the epicardial artery, may be a potential marker of PCI-induced improvement of total vascular resistance and microvascular decompression, while normalisation of wave speed from high to low may be an indicator of microvascular resistance attenuation. These indices require future outcome-linked studies to establish prognostic value.

### Peri-PCI changes in WIA in patients with CCS

We have demonstrated phase-specific distinct characteristics of peri-PCI improvement of cardiac–coronary coupling at rest and during hyperaemia in CCS (figure 1). Forward travelling waves, generated proximally and transmitted from the aorta to the distal coronary artery, seem to be primarily improved by PCI and not directly by microvascular vasodilation. Neither pre-PCI nor post-PCI hyperaemia causes a significant and consistent amplitude augmentation in FCW and FEW compared with rest. Therefore, the systolic coronary perfusion energetics appear not to be primarily hyperaemia-dependent but epicardial patency-dependent in the presence of a coronary narrowing. ‘Backward’ waves of microvascular origin are primarily affected by hyperaemic stimulation. This mechanism may indicate that the increased myocardial oxygen demand is mainly managed by wave energy augmentation during the expansion period, despite unaltered forward energy flux. Nonetheless, in the presence of a severe epicardial stenosis, microvascular decompression cannot sufficiently suction flow through the stenosis which causes an attenuation of BEW, before PCI, even when the microvascular vasodilation capacity seems strong—marked by high MRR. Our data demonstrate that hyperaemic stimulation causes a substantial augmentation of BCW magnitude compared with rest, both before and after PCI. However, resting BCW amplitudes did not significantly change following the relief of epicardial obstruction. BCW is generated by systolic compression of the microcirculation, it may be argued that its amplitude should be modulated by the state of microvascular vasodilation (which amplifies the displaced blood volume) rather than upstream epicardial conductance. While this observation aligns with the concept that BCW might serve as an epicardial disease-independent marker of myocardial contractility,^8^ our current dataset is not equipped to definitively prove this mechanism. Therefore, the exact mechanobiological drivers of BCW in the presence of epicardial disease remain a hypothesis that warrants validation in future experimental models. The described WIA characteristics are in line with what is seen following the unloading of left ventricle by transaortic valve replacement in a patient with aortic stenosis, where removal of the stenosis led to direct augmentation of FCW, FEW and BEW but not BCW at rest.^9^ However, in the same study, Rolandi *et al*,^9^ reported the lack of significant hyperaemic BEW augmentation, which is due to the relative hyperaemic state at rest and residual microvascular compression due to remaining left ventricular hypertrophy. Indeed, the hyperaemia caused substantial BCW amplification both before and after TAVI, consistent with our study. Importantly, all wave peaks occurred sooner (normalised for expansion and compression period length of patients), indicating normalisation of ventricular–coronary interaction delay of pre-PCI state.

In the context of obstructive CCS, there is a lack of comprehensive, robust knowledge regarding the coronary wave intensity characteristics. Only a few studies have so far interrogated WIA in stable obstructive CAD, which have only reported partial observations, selectively including some WIA peaks and/or not providing resting and hyperaemic measurements together or not comparing the paired pre-PCI and post-PCI states. These factors led to the apparently contradicting clinical observations.^1 2 9^ Narayan *et al* demonstrated an improvement of accelerating wave amplitudes following PCI^1^; however, they only reported the hyperaemic WIA amplitudes. In their study, pre- versus post-PCI values of hyperaemic BCW were comparable, whereas in our study, there was more than a 100% augmentation. Although their main observation regarding increased accelerating wave energy is concordant with the present observations, the lack of paired resting measurements pre- and post-PCI hinders a true comparison of the findings with their study. On the other hand, deMarchi *et al* demonstrated a significant temporary periprocedural augmentation of BEW during hyperaemia, and substantial reduction of BEW during 1-minute-long experimental occlusion of the proximal coronary artery by balloon inflation which is concordant with our observations indicating responsiveness of BEW to both epicardial conductance and microvascular vasodilation. Yet, they reported insignificant linear correlations between BEW and FFR, percentage of diameter stenosis and CFR, concluding that there is no correlation between epicardial disease severity and BEW in stable CAD.^2^ Despite their interpretation and conclusion, they have still demonstrated a significant (−10.120 W/m^2^/s, p<0.01) decrease in BEW during balloon-occlusion, which is in line with (given the temporal inverse) our pre- versus post-differences. Moreover, their study did not include post-PCI measurements.

### Relevance of WIA parameters to PCI-induced normalisation of epicardial and microvascular resistance

PCI amplified the accelerating wave energy as well as augmented the response to the hyperaemia, and improved the ventricular–coronary interaction delay. Increased resting BEW, marking the normalisation of microvascular suction that is responsible for coronary filling during the expansion period (and diastole), was associated with a reduction in total vascular resistance. The alternative expression of FFR as hMR/total resistance ratio may help in interpreting the demonstrated link.^10^ Mechanistically supported by the co-dependence of BEW on both epicardial resistance and microvascular vasodilation, BEW augmentation after PCI may provide a complementary physiological marker of haemodynamic success beyond pressure-only indices. In our cohort, physiological improvement was also reflected by large reductions in bSR, hSR and improved Pd/Pa and CFR, consistent with restoration of epicardial patency and improved downstream flow. ∆BEW also strongly correlated with hSR normalisation (∆hSR), a stenosis-specific index with established diagnostic robustness and prognostic value that may exceed FFR and/or iFR.^11–13^

Previous research suggested that epicardial lesions with severe impact on coronary flow are associated with ventricular–coronary interaction delay, that is defined by delayed peak impact of compression and/or decompression of myocardium and thus coronary circulation,^7^ and reported that the transfer of wave energy occurs sooner following removal of a stenosis in various settings including.^9 14^ Elective PCI seems to normalise the cardiac–coronary coupling in temporal axis in addition to improvement of magnitude of energy transfer. Incorporation of this temporal perspective may unravel subclinical improvement attributable to PCI, that may not be obvious in conventional flow/pressure metrics.

In addition, coronary wave speed—previously shown to be elevated distal to coronary stenoses and associated with local arterial wall properties—decreased after PCI at rest and during hyperaemia.^15 16^ However, this finding must be interpreted with caution. The sum-of-squares method assumes negligible correlation between forward and backward waves. Distal to a severe epicardial stenosis, prominent wave reflections could theoretically violate this assumption, which can cause the estimation method to break down and mathematically inflate the pre-PCI wave speed.^16^ Notably, a previous in vivo study by deMarchi *et al*^2^ failed to systematically observe these expected prominent reflections distal to stenoses, suggesting the in vivo impact of this theoretical limitation might vary. Therefore, the observed reduction in wave speed post-PCI (and its correlation with ∆hMR; r=0.77, p<0.001) may reflect a combination of actual physiological microvascular unloading and the restoration of the mathematical validity of the sum-of-squares method following the removal of the stenotic reflection source. Recognising the inherent challenges of separating waves in diseased coronaries, our study used net wave intensity parameters which are calculated independently of wave speed estimations. It is important to acknowledge the inherent mathematical coupling between wave intensity parameters, wave speed and classical resistance indices, which are all derived from the same simultaneous pressure and Doppler velocity signals over the same cardiac cycles, some degree of shared structure in their correlations is expected. However, the primary value of WIA does not rely on its strict mathematical independence from classical indices, but rather on its unique capacity to provide an integrating perspective. By combining pressure and flow with high temporal resolution, WIA extends beyond time-averaged bulk parameters to quantify the phase-dependent forces that establish and alter coronary flow throughout the cardiac cycle.

### Potential clinical implications: WIA to assess benefit and success of PCI

The prognostic impact and clinical benefit of PCI for stable, obstructive CCS has been widely discussed, particularly during and following the ORBITA and ISCHEMIA trial programmes.^17–19^ In this context, our findings provide mechanistic support that elective PCI can restore phasic energy transmission from the ventricle to the coronary circulation—reflected by coordinated changes in both forward and backward wave components—beyond improvements in pressure-only indices. While our physiological findings demonstrate that elective PCI significantly improves phasic cardiac–coronary coupling, the prognostic value of these WIA parameters—specifically resting BEW and coronary wave speed—was not tested in this study. Whether the normalisation of these mechanical forces translates into improved long-term clinical outcomes or symptom relief remains unknown and must be rigorously evaluated in future prospective, outcome-linked cohorts.

From a practical standpoint, WIA-derived metrics may have three potential applications: (1) mechanistic phenotyping of phasic ventricular–coronary coupling before and after PCI; (2) identification of residual micro-/macro-vascular dysfunction or impaired diastolic suction despite an apparently ‘successful’ PCI by FFR; and (3) use as physiological endpoints in future trials of PCI optimisation and microvascular therapies. At present, these applications remain investigational because WIA requires high-quality pressure–Doppler signals and outcome-based validation. Accordingly, prospective studies with standardised lesion characterisation, risk-factor stratification and clinical follow-up are needed.

### Limitations

This is a single-centre, retrospective, hypothesis-generating study in a relatively small number of patients and no a priori sample size calculation was performed. Lesion-level angiographic characteristics (vessel territory, lesion length, calcification, revascularisation history and lesion preparation strategies such as predilatation, postdilatation or atherectomy) were not systematically recorded, limiting inference on how morphology, prior PCI or lesion preparation influences wave mechanics. Although hypertension and diabetes, or medications, may influence arterial stiffness and wave speed, we did not have sufficiently granular patient-level covariate data linked to the physiology files to support robust subgroup analyses; this should be addressed in future prospective studies. No independent core laboratory was used; however, all signal processing and WIA were performed offline by investigators blinded to the PCI timepoint. Finally, clinical outcomes were not assessed; therefore, the prognostic value of BEW and wave speed after PCI requires validation in larger outcome-linked cohorts.

## Conclusion

PCI improves systolic and diastolic ventricular—coronary coupling associated with flow improvement under resting and pharmacologically simulated hyperaemic conditions by different phasic mechanisms, which may be specifically examined by WIA. PCI-induced normalisation of coronary epicardial resistance along with microvascular decompression (microvascular resistance) simultaneously occurs with improvement of WIA parameters.

## Data Availability

No data are available.
